# Locally deployed large language model for real‐world lecanemab eligibility pre‐screening

**DOI:** 10.1002/dad2.70495

**Published:** 2026-09-27

**Authors:** Carolin Miklitz, Maya Shrestha, Wegner Philipp, Nils Henk, Alois Martin Sprinkart, Wolfgang Block, Julian Alexander Luetkens, Alexander Radbruch, Nils Christian Lehnen, Sebastian Nowak

**Affiliations:** ^1^ Department of Old Age Psychiatry and Cognitive Disorders University Hospital Bonn Bonn Germany; ^2^ German Center for Neurodegenerative Diseases (DZNE Bonn) Bonn Germany; ^3^ Rhenish Friedrich Wilhelm University of Bonn Bonn Germany; ^4^ Department of Diagnostic and Interventional Radiology University Hospital Bonn Bonn Germany; ^5^ Department of Diagnostic and Interventional Neuroradiology and Pediatric Neuroradiology University Hospital Bonn Bonn Germany; ^6^ Department of Radiotherapy and Radiation Oncology University Hospital Bonn Bonn Germany

**Keywords:** Alzheimer's disease, disease‐modifying therapy, large language models, lecanemab, secure clinical AI deployment, treatment eligibility

## Abstract

**INTRODUCTION:**

The introduction of disease‐modifying Alzheimer's therapies requires complex, labor‐intensive patient screening. Cloud‐based large language models (LLMs) could support this task but are often unsuitable for routine care due to data protection constraints.

**METHODS:**

We evaluated an on‐premises, open‐weights LLM (gpt‐oss‐120b) for automated extraction of therapy‐relevant variables for lecanemab eligibility from German memory clinic reports. In a two‐stage design, LLM‐based extraction prompts, a deterministic rule‐based extractor, and a shared downstream rule‐based classifier were optimized on a development set (*n* = 97) and evaluated on an independent hold‐out set (*n* = 99), with expert consensus as ground truth.

**RESULTS:**

The LLM‐based pipeline achieved 94% accuracy and a Cohen's kappa of 0.90 on the hold‐out set, significantly surpassing the rule‐based comparator (80% accuracy) and demonstrating performance comparable to human experts.

**DISCUSSION:**

A locally deployed, on‐premises LLM may assist eligibility screening as a triage support tool, potentially facilitating access to novel therapies without compromising patient data privacy.

## Highlights

- A privacy‐preserving large language models (LLMs) classified lecanemab eligibility from clinical reports.
- Independent validation demonstrated 94% accuracy, exceeding a rule‐based comparator.
- Classification performance was comparable to individual expert rater assessments.
- On‐premises deployment ensured strong clinical data protection compliance.

## 1 BACKGROUND

The advent of disease‐modifying therapies represents a pivotal shift in treatment paradigms for early Alzheimer's disease (AD).^1^, ^2^ The monoclonal amyloid‐targeting antibodies lecanemab and donanemab have shown promise in reducing cerebral amyloid deposition and slowing cognitive decline.^3^, ^4^, ^5^ In the approval studies, both lecanemab and donanemab demonstrated a significant reduction in cerebral amyloid load as measured by amyloid positron emission tomography (PET), along with a reduction in cognitive deterioration during the 18‐month observation period. The most frequently observed side effects included cephalgias and amyloid‐related imaging abnormalities (ARIAs), which manifested as cerebral edema, micro‐ and macrohemorrhage.^6^, ^7^, ^8^, ^9^

With amyloid‐targeting therapies already approved for more than two year in the United States and several other countries,^10^, ^11^ both lecanemab (Leqembi®, April 2025) and more recently donanemab (Kisunla™, September 2025) have now received European Union (EU) marketing authorization for the treatment of early AD in individuals with confirmed amyloid pathology. Their use in the EU is restricted to apolipoprotein E (ApoE) ε4 non‐carriers or heterozygotes with early symptomatic AD and is worldwide guided by strict inclusion and exclusion criteria to minimize safety risks, particularly the occurrence of ARIA.^12^, ^13^, ^14^

Cummings et al. published Appropriate Use Recommendations (AUR) for treatment with lecanemab, outlining comprehensive inclusion and exclusion criteria to guide patient selection and risk mitigation.^15^ These recommendations have since been discussed and adapted from a European perspective to align with European Medicines Agency (EMA) labeling and healthcare structures.^16^, ^17^

Detailed clinical information about in‐ and exclusion criteria is often embedded within comprehensive medical reports that consolidate patient history, imaging and laboratory results, and clinical correspondence. Traditionally, the review of these reports is performed manually by the attending clinicians. This process is labor‐intensive and prone to inter‐rater variability and human error, especially when examining large patient cohorts using complex, multifactorial criteria.^18^

Large language models (LLMs) such as Generative Pre‐Trained Transformer 4 (GPT‐4, OpenAI) have been evaluated for numerous clinical tasks, including the generation of discharge summaries and the diagnosis of complex clinical cases, demonstrating promising results.^19^, ^20^, ^21^, ^22^ In AD, LLMs have been applied to electronic health records (EHRs) to identify disease phenotypes and extract diagnostic information from unstructured text: Knox et al. demonstrated large‐scale phenotyping of AD and related dementias using EHR data,^23^ Li et al. introduced a multi‐agent LLM framework for AD prediction from longitudinal clinical notes,^24^ and a recent GPT‐4o‐powered framework has been proposed for identifying cognitive impairment stages from EHRs.^25^

While such proprietary, cloud‐based LLMs have consistently achieved state‐of‐the‐art performance on medical reasoning tasks,^26^ their use remains constrained by data security, privacy, and regulatory concerns.^27^, ^28^, ^29^ A recent study demonstrated that open‐weights LLMs, which enable a fully Genereral Data Protection Regulation (GDPR, EU law) and Health Insurance Portability and Accountability Act (HIPAA, US law) compliant application on institutional infrastructures within secured clinical networks, can achieve performance parity with proprietary models for extracting information from free‐text radiology reports.^30^

In this study, we evaluate the open‐weights model gpt‐oss‐120b for a real‐world clinical natural language processing task: extracting eligibility‐relevant clinical features from narrative consultation reports in an outpatient memory clinic to support lecanemab screening. Focusing on patients with mild cognitive impairment (MCI) or mild dementia suspected due to AD, we employed a rigorous two‐stage study design separating prompt development from independent validation. In the development stage, both the LLM information extraction prompts and a downstream rule‐based classification logic were iteratively optimized. To assess the added value of the LLM, we also developed a deterministic rule‐based extraction pipeline that served as a non‐LLM comparator while using the same downstream eligibility classifier. In the validation stage, the finalized LLM‐assisted pipeline and the rule‐based comparator were applied to a temporally independent hold‐out test set to classify participants as eligible, potentially eligible, or not eligible. The main objective was to determine whether a locally deployed, on‐premises, LLM‐assisted eligibility pipeline can reliably assist screening as a triage support tool, achieving expert‐level agreement, while preserving strict data privacy requirements. A secondary objective was the comparison of its performance with a deterministic rule based extraction approach, thereby evaluating the added value of the LLM‐based information extraction.

**RESEARCH IN CONTEXT**

1. **Systematic review**: A PubMed search identified studies investigating the use of large language models (LLMs) in dementia diagnostics and clinical care. Early work has demonstrated promising results for Alzheimer's disease phenotyping and staging based on clinical text. However, evidence on locally deployable LLM solutions supporting therapy‐related workflows and real‐world clinical implementation remains limited.
2. **Interpretation**: This study demonstrates the feasibility of a locally deployed, LLM‐assisted pipeline for determining lecanemab eligibility from routine clinical reports. The system achieved high agreement with a consensus reference standard in a memory clinic setting, performed comparably to individual expert raters, and outperformed a deterministic rule‐based comparator. These findings support its use in identifying eligible patients, facilitating trial pre‐screening, improving consistency of eligibility assessments within clinician‐supervised workflows, while enabling secure on‐premises deployment.
3. **Future directions**: Future studies should evaluate prospective integration into clinical workflows for real‐time clinical decision support and validate performance across independent multicenter cohorts.

## 2 METHODS

### 2.1 Study design and ethics

The study followed a two‐stage design comprising iterative development and independent hold‐out evaluation and included two information extraction approaches: an LLM‐based pipeline and a deterministic rule‐based information extraction pipeline serving as a non‐LLM comparator. Both extraction approaches used the same downstream eligibility classifier. During the development stage, a dataset of *n* = 97 medical consultation reports was used to iteratively optimize the LLM prompts, the rule‐based extraction pipeline, and the downstream classification logic to maximize agreement with the reference standard. In the second stage, the finalized systems were evaluated on a strict hold‐out test set of *n* = 99 temporally and patient‐wise distinct consultation reports. The hold‐out set was accessed only after the prompts, extraction pipeline, and classification logic had been finalized; no further modifications were made based on these cases. The study was conducted in accordance with the ethical principles of the Declaration of Helsinki. The study was approved by the Ethics Commission of the University of Bonn (ethics approval number: 312‐23‐EP), and the requirement for written informed consent was waived.

### 2.2 Study sample

**FIGURE 1:**
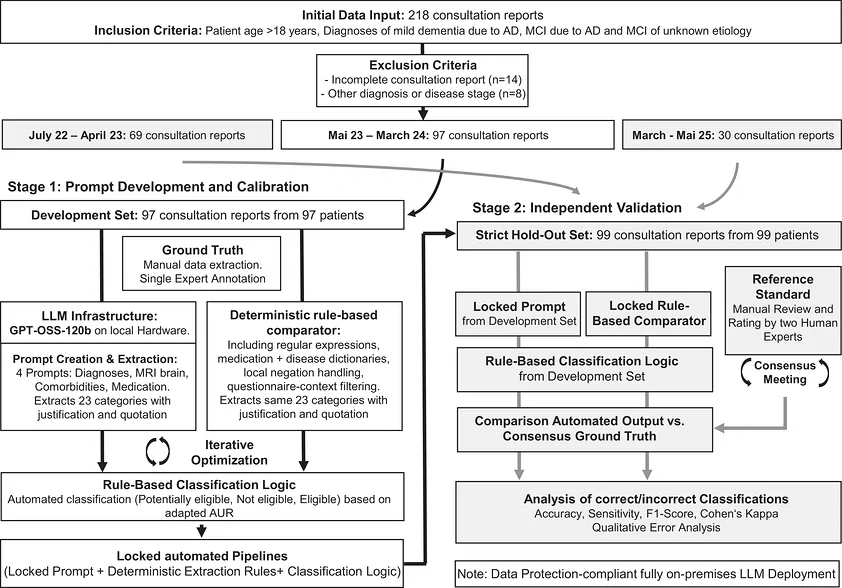
Flowchart of patient inclusion and exclusion criteria, data analysis, and generation of results. The study comprised two information extraction approaches: an LLM‐based pipeline and a deterministic rule‐based information extraction pipeline serving as a non‐LLM comparator, both using the same downstream eligibility classifier. In the development phase, modular LLM prompts, the rule‐based extraction pipeline, and the downstream classification logic classification (Python‐based logic with three categories: potentially eligible, eligible, and not eligible) were optimized on 97 consultation reports to maximize agreement with the reference standard. In the independent validation phase, the finalized automated pipelines were applied to new consultations reports (*n* = 99) to generate final category assignments, which were compared with a two‐expert reference standard. The hold‐out test set was temporally and patient‐wise distinct and was accessed only after all system components had been finalized. AD, dementia due to Alzheimer's disease; LLM, Large language model; MCI, mild cognitive impairment.

The study included 196 medical consultation reports from 196 patients presenting at the outpatient memory clinic of the University Hospital Bonn. The development set (*n* = 97) consisted of patients presenting for final consultation after diagnostic dementia assessment from May 2023 ‐ March 2024. The hold‐out data set (*n* = 99) comprised two independent cohorts that were not used during development: an earlier retrospective cohort (July 2022 ‐ April 2023, *n* = 69) and a later temporal hold‐out cohort (March–May 2025, *n* = 30). The consultation reports were generated during a period in which anti‐amyloid therapies had not yet been implemented as part of routine clinical care at the study center. Consequently, the reports did not contain an explicit assessment of lecanemab eligibility, and eligibility‐relevant information had to be extracted from routine narrative clinical documentation. For both the development and the hold‐out data set, inclusion criteria were: (1) age > 18 years, and (2) a clinically or biomarker‐confirmed diagnosis of mild dementia due to AD), MCI due to AD, or MCI of unclear etiology. Exclusion criteria included diagnoses of non‐AD dementia forms, moderate to severe dementia stages, subjective cognitive decline, or suspected non‐AD MCI as well as incomplete medical documentation. Figure 1 presents a flow chart of the study design and the participant selection.

### 2.3 Input data

The input of the model consisted of free‐text medical consultation reports following a clinical template, including sections on diagnosis, anamnesis, psychopathological finding, physical examination, additional examinations (e.g., cerebrospinal fluid analysis and textual MRI reports), and clinical course. The model did not process or interpret raw MRI images. No additional structured data (e.g., demographic variables, coded diagnoses or laboratory values outside the report text) were provided to the model.

### 2.4 Eligibility classification

Eligibility was classified following the inclusion and exclusion criteria outlined in the AUR and adapted for European contexts (Table 1), which were applied consistently across clinical assessments and the automated pipeline. Eligibility was categorized into three classes: not eligible, potentially eligible, and eligible. *Not eligible*: Assigned if any strict exclusion criterion was met (e.g., MMSE < 22, history of stroke/epilepsy, excluding MRI findings like macro‐hemorrhage or superficial siderosis, or use of anticoagulants/immunosuppressants). *Potentially eligible*: Assigned if no exclusion criteria were found, but essential data required for a definitive eligible decision were missing (e.g., missing amyloid status, missing MMSE score, or missing MRI report). *Eligible*: Assigned only if all inclusion criteria were confirmed and no exclusion criteria were present.

**TABLE 1:** Eligibility criteria and contraindications for treatment with lecanemab.

Category	Eligibility criteria
Diagnosis	Mild cognitive impairment or mild dementia due to AD
Amyloid biomarker	CSF Aß42 and/or Aß42/40 ratio positive or amyloid PET positive
MMSE	≥ 22
MRI	MRI availability

Category	Contraindications
MRI	MRI signs of haemorrhage
	MRI signs of superficial siderosis
	MRI signs of cerebral ischaemia
	Fazekas Score 3
	MRI signs of Cerebral Amyloid Angiopathy
	MRI signs of non‐AD dementia
Comorbidity	Transient ischemic attack or stroke within the past 12 months
	Epileptic seizure within the past 12 months
	Major depressive episode
	Immunological disease
	Bleeding disorder
Medication	Immunosuppressive medication
	Anticoagulation

Abbreviation: Aß, amyloid‐beta; AD, Alzheimer's disease; CSF, cerebrospinal fluid; MMSE, Mini‐Mental Status Examination; MRI, magnetic resonance imaging; PET, positron emission tomography.

### 2.5 Ground truth generation

The development set was annotated by a single trained rater (rater 0), who extracted all relevant clinical features to determine eligibility, with uncertain cases resolved via senior physician consultation. To establish a reference standard for evaluation, a rigorous multi‐rater process was implemented for the hold‐out test set. Two experienced clinicians (rater 1 and rater 2) independently reviewed the 99 medical records and determined eligibility according to the predefined classification rules. Discrepancies between the two human raters were reviewed and resolved in a consensus meeting by the raters. The resulting consensus rating served as the ground truth reference for the performance comparisons.

### 2.6 LLM infrastructure and experimental setup

We utilized gpt‐oss‐120b, an open‐weights LLM released by OpenAI (August 2025), deployed entirely on‐premises within the hospital's secure clinical network to ensure strict data privacy and data protection compliance. The inference pipeline was orchestrated using the open‐source engine SGLang (v0.5.3.post1). To prevent hallucinations and ensure syntactic validity, we employed XGrammar structured decoding, which enforced a predefined JSON schema for the model's output. The extraction task was decomposed into four modular steps: (1) diagnosis and cognition, (2) MRI findings, (3) comorbidities and anamnesis, and (4) systemic diseases and medication (Table S1 and Table S2). For each extracted data point, the model was required to provide the extracted value, a reasoning summary, and a direct verbatim quote from the source text. Prompts were structured to define the model's role, specify extraction tasks, and enforce a standardized output format. Prompts were developed and optimized exclusively on the development set based on observed extraction and classification errors, were finalized prior to evaluation and not modified thereafter. Final performance was evaluated on the independent hold‐out test set.

### 2.7 Deterministic rule‐based comparator

To assess the added value of a LLM‐based feature extraction pipeline, we implemented a deterministic rule‐based comparator. The comparator used pre‐specified regular expressions, small medication and disease dictionaries, local negation handling, family‐history filtering, questionnaire‐context filtering, and limited numeric parsing for amyloid biomarkers to extract the same eligibility‐relevant clinical variables from consultation reports as the LLM. The extracted variables were subsequently processed using the identical downstream rule‐based eligibility classifier. A detailed description of the development, extraction rules and implementation of the rule‐based extraction algorithm is provided in Supplementary Methods 1.

### 2.8 Automated classification logic

A rule‐based Python classification system mapped the structured output of the LLM and the rule‐based comparator to the three eligibility classes according to the predefined criteria outlined above. To ensure consistent and reproducible implementation, the classification logic was iteratively refined during the development phase through detailed operationalization of the eligibility criteria. To reflect clinical reality, configurable whitelists were implemented during the development phase to permit specific non‐excluding conditions (e.g., “cerebral microangiopathy” with Fazekas score < 3) and medications (e.g., “topical immunosuppressants”).

### 2.9 Statistical evaluation

The primary endpoint was the performance of the LLM‐based classification system on the hold‐out test set (*n* = 99) compared to the human consensus ground truth. The secondary endpoint was the added value of LLM‐based information extraction compared with a deterministic rule‐based approach using the same downstream eligibility classifier. Performance was evaluated using accuracy, weighted F1‐score, and Cohen's kappa. For each eligibility class, recall (sensitivity), precision (positive predictive value [PPV]), and F1‐score were also reported. In an additional case‐finding analysis, eligible and potentially eligible categories were combined as the positive class, and sensitivity, specificity, PPV, and negative predictive value (NPV) were calculated. Where appropriate, 95% confidence intervals were estimated using 1,000 case‐level bootstrap resamples. The LLM‐based pipeline and rule‐based comparator were compared on paired hold‐out cases using an exact two‐sided McNemar test, with confidence intervals for paired performance differences estimated by 1,000 paired bootstrap resamples.

To contextualize model performance, inter‐rater agreement between the two clinicians was assessed using Cohen's kappa. Agreement between individual raters and the consensus was reported descriptively, acknowledging that these comparisons were not fully independent. Model performance was interpreted relative to the range of inter‐rater variability among human experts. Disagreement analysis was performed both in the development and the hold‐out test set for any cases where the LLM‐based eligibility pipeline or the rule‐based non‐LLM classification deviated from the consensus.

## 3 RESULTS

### 3.1 Study sample

A total of 196 consultation reports were processed across the two study stages. The development set included 97 patients (mean age 73.9 ± 8.2 years; 66 females, 31 males, May 2023–March 2024) and was used for iterative optimization of the LLM prompts, the deterministic rule‐based comparator, and the downstream classification logic. The independent hold‐out test set consisted of 99 consultation reports from 99 patients (mean age 73.6 ± 8.6 years; 57 females, 42 males) from two different time periods (July 2022 to April 2023 and March 2025 to May 2025) who were not included in the development set. All records in the development and the hold‐out test set were successfully processed by gpt‐oss‐120b and a deterministic rule‐based comparator. Patient characteristics and eligibility status for the development and the hold‐out test set are summarized in Table 2.

**TABLE 2:** Patient characteristics and eligibility status in different categories.

	Development set (*N* = 97)	Hold‐out test set (*N* = 99)
Category	Number (%)	Number (%)
Diagnosis		
Mild dementia	54 (55.7%)	40 (40.4%)
Mild cognitive impairment	42 (43.3%)	53 (53.5%)
Dementia, unknown severity	1 (1.0%)	6 (6.1%)
Age		
Mean (SD)	73.9 (8.21)	73.6 (8.57)
Sex		
Female	66 (68.0%)	57 (57.6%)
Male	31 (32.0%)	42 (42.4%)
Eligibility criteria lecanemab		
Biomarker		
Amyloid positive	44 (45.4%)	51 (51.5%)
Amyloid negative	8 (8.2%)	6 (6.1%)
Missing	45 (46.4%)	42 (42.4%)
MMSE ≥22		
< 22	15 (15.5%)	10 (10.1%)
≥22	66 (68.0%)	84 (84.8%)
Missing	16 (16.5%)	5 (5.1%)
Contraindication MRI		
Contraindication	5 (5.2%)	6 (6.1%)
No contraindication	74 (76.3%)	87 (87.8%)
Missing	18 (18.6%)	6 (6.1%)
Contraindication comorbidity		
Contraindication	14 (14.4%)	9 (9.1%)
No contraindication	83 (85.6%)	90 (90.9%)
Contraindication medication		
Contraindication	16 (16.5%)	14 (14.1%)
No contraindication	81 (83.5%)	85 (85.9%)

Abbreviation: MMSE, Mini‐Mental State Examination; MRI, magnetic resonance imaging; SD, standard deviation.

### 3.2 Performance on development set

Based on manual human expert extraction, in the development set 19 participants (19.6%) were classified as eligible for lecanemab treatment, 33 (34.0%) as potentially eligible, and 45 (46.4%) as not eligible. The LLM‐assisted pipeline classified 20 (20.6%), 30 (30.9%), and 47 (48.5%) participants into the respective categories, achieving an accuracy of 0.959 (95% confidence interval [CI] 0.918‐0.990), a weighted F1‐score of 0.959 (95% CI 0.915–0.990), and a Cohen's kappa of 0.935 (95% CI 0.861–0.984). The deterministic rule‐based comparator classified 18 (18.6%), 35 (36.1%), and 44 (45.4%) participants into the respective categories, achieving an accuracy of 0.897 (95% CI 0.835–0.948), a weighted F1‐score of 0.898 (95% CI 0.837–0.949), and a Cohen's kappa of 0.836 (95% CI 0.733–0.919). As both pipelines were optimized on the development set, these results are descriptive rather than confirmatory. Confusion matrices are shown in Figure S1.

Both systems generated separate outputs (extractions, rationales, and citations) that facilitated analysis of disagreement cases. Examples of these output files are presented in Supplementary Appendix 1. Analysis of the four discordant development cases from the LLM‐based pipeline identified three error patterns that informed prompt optimization: conservative interpretation of ambiguous psychiatric symptoms, medication misclassification, and differences in the operational interpretation of MRI ischemia criteria. Overall, these findings indicated a conservative, safety‐oriented extraction strategy.

### 3.3 Performance on independent hold‐out test set

#### 3.3.1 LLM‐based pipeline

**FIGURE 2:**
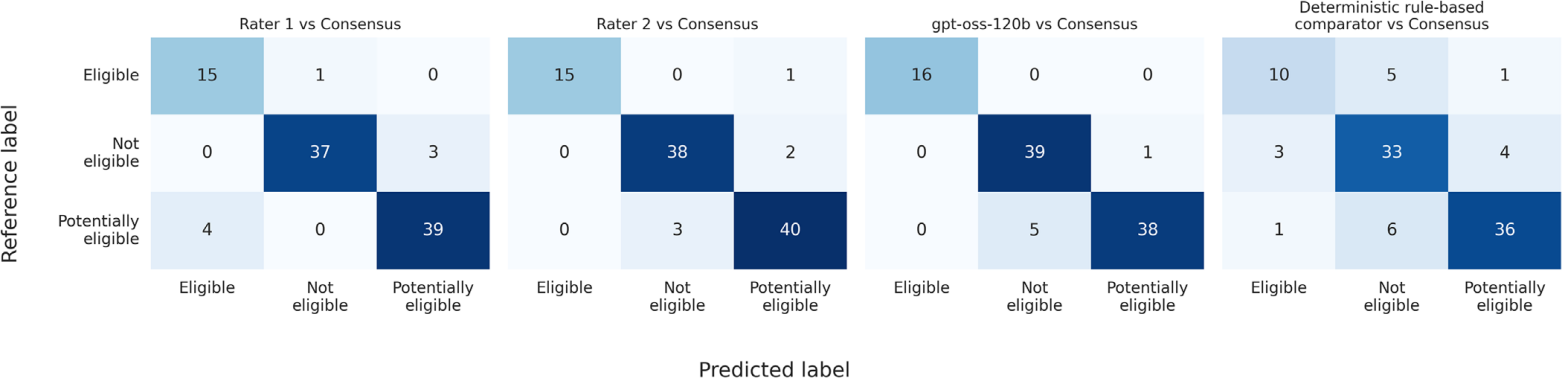
Confusion matrices comparing two human raters, the large language model, and the deterministic rule‐based comparator versus human consensus on the hold‐out test set. From left to right, classifications assigned by rater 1, rater 2, the gpt‐oss‐120b pipeline, and the deterministic rule‐based comparator (predicted labels) are compared with human‐consensus labels (true labels) on the hold‐out test set (*n* = 99). Cells along the main diagonal represent matching classifications.

**FIGURE 3:**
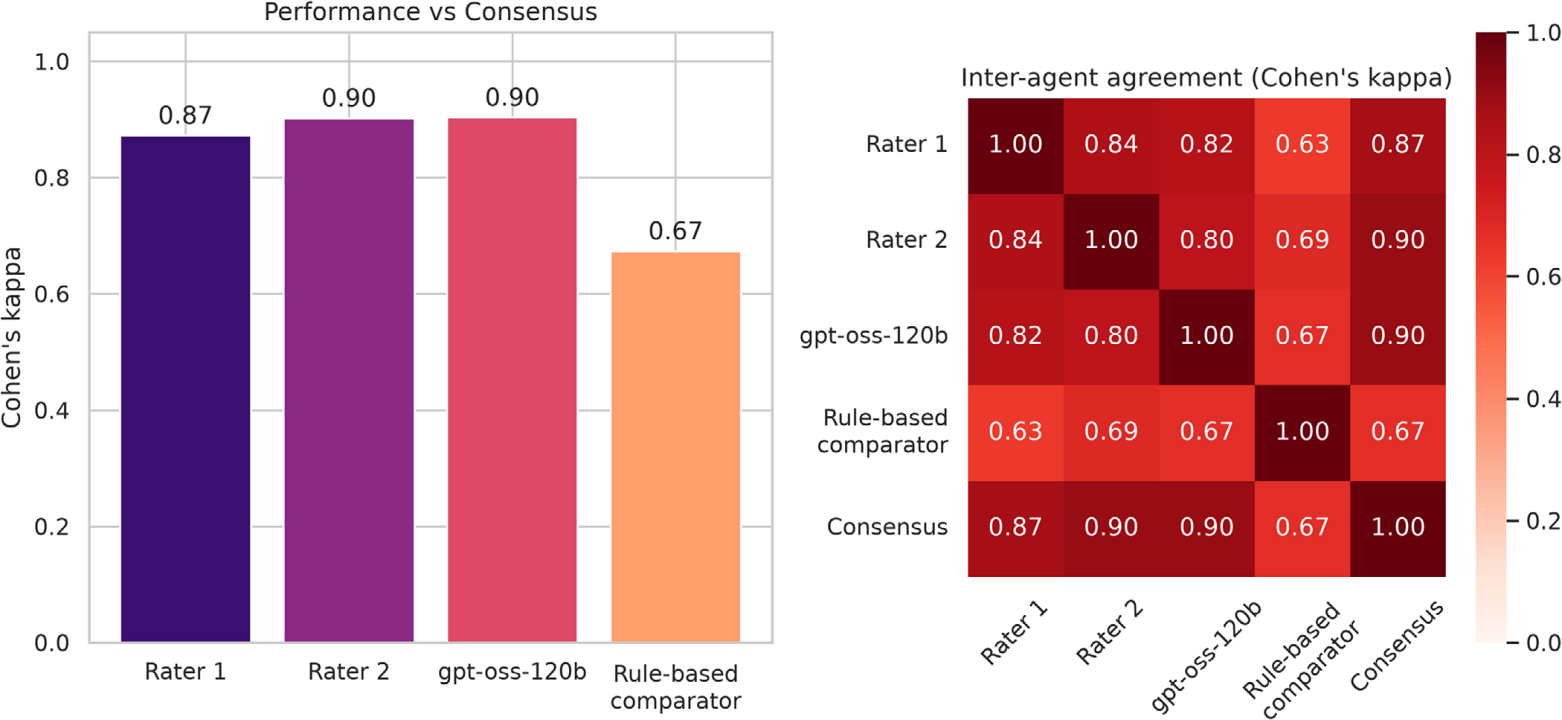
Comparative performance and inter‐rater reliability between the two experts (rater 1, rater 2), the classifications based on the gpt‐oss‐120b pipeline, and the deterministic rule‐based comparator on the hold‐out test set (*n* = 99). (Left) Bar chart of Cohen's kappa of agreement with the clinical consensus ground truth. (Right) Pairwise inter‐rater reliability heatmap based on Cohen's kappa.

To contextualize the pipeline's performance relative to clinical practice, the automated system was evaluated against the consensus reference standard and interpreted in relation to the performance of two individual human experts (Figures 2 and 3). According to clinical consensus, in the hold‐out test set 16 persons (16.2%) fulfilled eligibility criteria for lecanemab treatment, 43 (43.4%) were classified as potentially eligible and 40 (40.4%) as not eligible. The LLM‐based pipeline classified 16 persons (16.2%) as eligible, 39 (39.4%) as potentially eligible and 44 (44.4%) as not eligible. Across the independent hold‐out test set, the LLM‐based pipeline demonstrated high agreement with the clinical consensus reference standard, with an accuracy of 0.939 (95% CI 0.889–0.980) and Cohen's kappa of 0.903 (95% CI 0.810–0.968). This level of agreement was within the range observed between human raters, with rater 1 achieving an accuracy of 0.919 (95% CI 0.868–0.970) and Cohen's kappa of 0.872 (95% CI 0.782–0.950), and rater 2 achieving an accuracy of 0.939 (95% CI 0.889–0.980) and Cohen's kappa of 0.902 (95% CI 0.820–0.968) (Figure 3, left). As illustrated in Figure 3 (right), inter‐rater reliability analyses further demonstrated that the LLM system's agreement with each human reviewer (kappa = 0.82 with rater 1; kappa = 0.80 with rater 2) was comparable to the agreement observed between the human raters themselves (kappa = 0.84). Overall performance, class‐specific performance, and case‐finding metrics are summarized in Table 3. Disagreements among human raters primarily involved subtle differences in the interpretation of MRI exclusion criteria (e.g., the operationalization of a “post‐ischemic residuum”), as well as oversight of missing data (e.g., a missing Mini‐Mental State Examination [MMSE] score) and exclusion‐relevant comorbidities (e.g., Crohn's disease and Graves' disease). Disagreement of the LLM‐based, automated system was observed in six cases. Errors included incorrect medication and disease categorization (beclometasone, psoriasis), context misinterpretation (differential diagnosis interpreted as main etiology), operational definition mismatch (depression severity), and clinical threshold misapplication (Fazekas grade 1, Table S3). In this specific test sample, the LLM‐based classification pipeline categorized cases with consistency and accuracy comparable to each of the two human reviewers.

**TABLE 3:** Performance of the LLM‐assisted eligibility pipeline, deterministic rule‐based comparator, and individual expert raters on the independent hold‐out test set.

System	gpt‐oss‐120b	Deterministic rule‐based comparator	Expert 1	Expert 2
Overall performance				
Accuracy (95% CI)	0.939 (0.889–0.980)	0.798 (0.717–0.869)	0.919 (0.868–0.970)	0.939 (0.889–0.980)
Weighted F1 (95% CI)	0.939 (0.887–0.980)	0.797 (0.718–0.870)	0.920 (0.868–0.970)	0.940 (0.889–0.980)
Cohen's kappa (95% CI)	0.903 (0.810–0.968)	0.673 (0.545–0.790)	0.872 (0.782–0.950)	0.902 (0.8200–.968)
Class‐specific performance				
Eligible				
Sensitivity (95% CI)	1.000 (1.000–1.000)	0.625 (0.364–0.857)	–	–
Precision/PPV (95% CI)	1.000 (1.000–1.000)	0.714 (0.454–0.929)	–	–
F1 (95% CI)	1.000 (1.000–1.000)	0.667 (0.421–0.828)	–	–
Not eligible				
Sensitivity (95% CI)	0.975 (.911–1.000)	0.825 (0.703–0.931)	–	–
Precision/PPV (95% CI)	0.886 (0.784–0.974)	0.750 (0.615–0.875)	–	–
F1 (95% CI)	0.929 (0.864–0.977)	0.786 (0.682–0.872)	–	–
Potentially eligible				
Sensitivity (95% CI)	0.884 (0.778–0.976)	0.837 (0.719–0.944)	–	–
Precision/PPV (95% CI)	0.974 (.912–1.000)	0.878 (0.771–0.973)	–	–
F1 (95% CI)	0.927 (0.861–0.976)	0.857 (0.769–0.929)	–	–
Case‐finding (combined eligible and potentially eligible)				
Sensitivity (95% CI)	0.915 (0.831–0.983)	0.814 (0.709–0.911)	–	–
Precision/PPV (95% CI)	0.982 (0.939–1.000)	0.873 (0.780–0.951)	–	–
Specificity (95% CI)	0.975 (0.911–1.000)	0.825 (0.703–0.931)	–	–
NPV (95% CI)	0.886 (0.784–0.974)	0.750 (0.615–0.875)	–	–

*Note*: Overall classification performance is reported as accuracy, weighted F1‐score, and Cohen's kappa with 95% confidence intervals (95% CIs). Class‐specific sensitivity, precision (positive predictive value (PPV)), and F1‐score are shown for the eligible, potentially eligible, and not eligible categories. Case‐finding performance was evaluated by combining the eligible and potentially eligible categories as the positive class, with sensitivity, PPV, specificity, and negative predictive value (NPV) reported. Confidence intervals were estimated using 1,000 case‐level bootstrap resamples.

#### 3.3.2 Deterministic rule‐based comparator

Based on the data entries extracted by the rule‐based, non‐LLM baseline 14 persons (14.1%) were classified as eligible, 41 (41.4%) as potentially eligible and 44 (44.4%) as not eligible (Figure 2). In the independent hold‐out set, the rule‐based comparator achieved an accuracy of 0.798 (95% CI 0.717–0.869) and Cohen's kappa of 0.673 (95% CI 0.545–0.790). Detailed performance metrics are provided in Table 3. Overall, performance was significantly lower than that of the LLM‐based pipeline (paired accuracy difference 0.141 [95% CI 0.061–0.222]; exact two‐sided McNemar *p* = 0.0026). Likewise, agreement between the rule‐based comparator and each human rater (kappa = 0.63 and 0.69) was numerically lower than both the inter‐rater agreement (kappa = 0.84) and the agreement between the LLM‐based system and each human rater (kappa = 0.82 and 0.80; Figure 3).

The 20 cases misclassified by the rule‐based extractor included errors involving linguistically variable or context‐dependent formulations of negated findings (e.g., phrasings indicating the absence of haemorrhage, infarction, or ischaemia), contextual misinterpretation (differential diagnoses incorrectly treated as confirmed diagnoses), information omission and failure to correctly distinguish measured amyloid biomarker values from laboratory reference ranges, leading to incorrect amyloid classification. A detailed overview of the misclassified cases for both the LLM‐based and rule‐based extraction approaches is provided in Table S3.

## 4 DISCUSSION

This retrospective study aimed to evaluate a locally deployed, on‐premises LLM for the automated extraction of eligibility‐relevant clinical features for lecanemab treatment from narrative consultation reports of patients with mild cognitive impairment or mild dementia due to AD, and to determine overall treatment eligibility based on the extracted information. This study assessed the feasibility and performance of a LLM‐assisted eligibility pipeline comprising information extraction and rule‐based classification, with strict separation of prompt development and validation using a dedicated hold‐out test set. Performance was additionally compared with an otherwise identical pipeline using a rule‐based extraction approach.

Our results indicate that the locally deployed LLM gpt‐oss‐120b can accurately capture clinically relevant information required for adjudication of lecanemab eligibility in a real‐world outpatient memory clinic setting. Based on these extracted data, the automated eligibility pipeline achieved high agreement with the consensus standard in this clinically complex decision task and significantly higher performance than the rule‐based comparator. In the independent hold‐out test set, the pipeline reached an accuracy of 0.94 and a Cohen's kappa of 0.90, exceeding the rule‐based comparator (accuracy 0.80, kappa = 0.67) and falling within the range of agreement observed among human raters (rater 1: accuracy 0.92 and kappa = 0.87, rater 2: accuracy 0.94 and kappa = 0.90). The analysis of human discrepancies revealed that experts occasionally overlooked missing data points or interpreted subtle radiological findings variably.

Comparison of the error profiles suggested that the higher performance of the LLM‐based pipeline primarily reflected its ability to robustly extract clinically relevant information from heterogeneous consultation reports. Whereas the rule‐based comparator was susceptible when negation, contextual qualifiers, and implicit information were expressed in linguistically variable or context‐dependent formulations, the few remaining LLM errors mainly reflected differences in the application of eligibility criteria rather than failures of information extraction. Consistent with this, the development‐to‐hold‐out decrease in accuracy was numerically larger for the rule‐based comparator (0.90 to 0.80) than for the LLM pipeline (0.96 to 0.94), suggesting weaker generalization of manually engineered rules to unseen linguistic variants.

The consistent application of predefined extraction and classification rules suggests that the LLM‐based pipeline may improve the consistency of eligibility assessments within clinician‐supervised workflows. In routine memory clinic practice, it could serve as a triage or pre‐screening tool to identify potentially eligible patients requiring further expert review. In addition, it may function as a secondary review tool to reduce eligibility errors arising from human fatigue or attentional lapses and to identify potential omissions or inconsistencies in complex eligibility determinations. The pipeline generated transparent structured outputs with explicit reasoning steps and source citations, enabling clinicians to review the underlying evidence for each decision. This transparency may facilitate its use as a clinician‐supervised triage and secondary review tool.

Notably, the LLM adopted a conservative extraction strategy, preferentially flagging potentially relevant exclusion criteria in ambiguous cases (e.g. interpretation of psoriasis as immunological disorder or a Geriatric Depression Scale (GDS) score of 13 as major depressive episode). As the pipeline is intended to support rather than replace clinical decision‐making, this conservative bias represents a desirable safety feature: extracted findings can be readily verified by clinicians using the accompanying source citations, whereas missed exclusion criteria remain unavailable for review. Consistent with this design, the pipeline achieved high case‐finding sensitivity (0.92) while maintaining excellent precision (PPV 0.98) for identifying eligible or potentially eligible patients.

A key design feature of the proposed pipeline is its three‐tier eligibility classification, which reflects the incomplete diagnostic work‐up frequently encountered in routine memory clinic practice. The “potentially eligible” category identifies persons who may benefit from additional diagnostic evaluation (e.g., amyloid biomarker assessment) before treatment eligibility can be determined, whereas persons with documented contraindications are classified as “not eligible” even if other information remains unavailable. This approach supports systematic pre‐screening, while definitive eligibility assessment requires completion of the recommended diagnostic work‐up and expert clinical review.

Our results align with and exceed recent benchmarks in the field. While previous studies using cloud‐based models like GPT‐4o (OpenAI) have reported strong agreement for cognitive staging (Cohen's kappa 0.95) ^25^ and locally deployed models like Me‐LLama have shown promising accuracy (∼86%) for dementia phenotyping,^23^ our system achieved high performance comparable to human experts on an independent hold‐out test set. This supports the hypothesis that particularly when processing protected health information in clinical settings, open‐weights models may play an important role in the future of AI‐assisted healthcare.^31^ Crucially, we accomplished this without external transmission of sensitive patient data, addressing a central data‐protection requirement that currently hinders the clinical integration of LLMs across Europe and the United States.^32^, ^33^

Consistent with previous epidemiological data, our study confirmed that only a minority of persons presenting at an outpatient memory clinic are eligible for lecanemab treatment.^34^, ^35^ In our total sample, approximately 18% of persons with mild cognitive impairment or mild dementia due to AD met the strict AUR‐aligned eligibility criteria. The observed inclusion rate should not be interpreted as precise estimate of real‐world eligibility, as current prescribing information^36^, ^37^ are less restrictive than the initial AUR and APOE‐ε4 status was missing from our data set. Nevertheless, the data demonstrate that the vast majority of patients with early dementia is not eligible for lecanemab treatment. This underscores the necessity for automated screening tools supporting clinicians in effectively identifying the small subset of patients who may benefit from lecanemab treatment, while ensuring guidance to alternative care pathways for non‐eligible patients.^38^

Notwithstanding the promising results, several limitations must be acknowledged. First, the study's retrospective nature and the monocentric study design limit the generalizability of the findings. Variability in clinical documentation across institutions may affect model performance, highlighting the need for prospective multicenter validation. Second, the study utilized German consultation reports. While the prompt structure is language‐agnostic, the specific phrasing of German clinical documentation may have influenced performance, and validation in other languages is warranted. Third, the model's performance depends on the quality of the source text, and the system cannot derive clinical facts that are not documented. Successful artificial intelligence implementation, therefore, hinges on parallel improvements in structured clinical documentation. Forth, this study focused on patients from MCI to mild dementia in a memory clinic setting; validation in larger and more diverse populations across disease severities, centers and care settings is necessary. Fifth, the present study demonstrated the feasibility and performance of the pipeline in a clinically relevant retrospective screening setting. Future studies should evaluate its incremental value in prospective routine clinical workflows for anti‐amyloid therapy. Finally, gpt‐oss‐120b is not a certified medical device. Its use in clinical practice requires regulatory approval and human‐in‐the‐loop supervision.

In conclusion, this study demonstrates the feasibility of a locally deployed, open‐source large language model‐based pipeline for determining lecanemab eligibility from routine clinical reports. The system achieved high agreement with the consensus reference standard, with performance comparable to that observed among human experts while exceeding that of the deterministic rule‐based comparator. Accordingly, this LLM‐based approach can serve as a triage support tool within clinician‐supervised workflows, enabling efficient identification of patients potentially eligible for treatment, facilitating clinical trial pre‐screening, and providing a secondary consistency check in complex eligibility determinations. Future work should focus on validation in larger and more diverse cohorts and on evaluating integration into clinical information systems to support real‐world clinical workflows.

## AUTHOR CONTRIBUTIONS

C.M. designed the study, conducted the primary analyses, and drafted the manuscript. S.N. led the data analysis and contributed substantially to conceptualization, methodological development, LLM implementation, manuscript preparation, and experiment supervision. M.S. and N.H supported data preparation and provided clinical expertise. P.W. supported data preparation and provided methodological expertise. N.C.L. contributed substantially to study design. N.C.L, A.R. and J.A.L. offered oversight and medical supervision. W.B. and A.M.S. offered oversight and technical supervision. All authors reviewed and approved the final manuscript.

## CONFLICT OF INTEREST STATEMENT

The authors declare no conflicts of interest related to the present study. Author disclosures are available in the Supporting Information.

## CODE AVAILABILITY STATEMENT

The code used for the experiments will be publicly available under https://github.com/ukb‐rad‐cfqiai/llm‐lecanemab‐eligibility upon publication of the manuscript.

## CONSENT STATEMENT

The study was conducted according to the Declaration of Helsinki. The study was approved by the Ethics Commission of the University of Bonn (ethics approval number: 312‐23‐EP), and the requirement for written informed consent was waived.

## Supporting information



Supporting Information

Supporting Information

Supporting Information

Supporting Information

Supporting Information

Supporting Information

Supporting Information

## Data Availability

The datasets analyzed during the current study are not publicly available because the source material consists of personal medical documents that cannot be fully anonymized without compromising their integrity. Data may be available from the corresponding author upon reasonable request, subject to appropriate privacy and ethical safeguards.
