# Supplementary material for: Locally deployed large language model for real‐world lecanemab eligibility pre‐screening
Source: Alzheimers Dement (Amst). 2026 Sep 27;18(3):e70495. doi: 10.1002/dad2.70495 (PMC13616205; doi:10.1002/dad2.70495)
Supplement: Supplementary file 1 [file DAD2-18-e70495-s001.pdf]

Supplementary Appendix 1. Example of System Output File facilitating Disagreement Analysis (translated from German)

ID 86

Analysis of Disagreement:

Ground Truth: *potentially eligible*
LLM based Classification: *not eligible*
Reasoning for LLM-based result: "Inadmissible immunosuppressive medication found: ['Cortisone']"

Complete LLM Extractions for this patient:

Extraction Block: Comorbidities and Anamnesis

- history_stroke: missing - LLM Reasoning: Neither a stroke is mentioned nor ruled out in the text, so the topic is missing. - Citation: *Text contains no information*
- history_epileptic_seizure: missing - LLM Reasoning: The text contains no indication of an epileptic seizure whatsoever, neither positive nor negative, therefore the information is missing. - Citation: *Text contains no information*
- depression: no - LLM Reasoning: It is expressly stated that no depressive symptoms are present, thus depression is ruled out. - Citation: *Indications of depressive or delusional symptoms do not arise.*
- depression_diagnosis_mention_context: [] - LLM Reasoning: Since depression is negated, there is no context of a depressive diagnosis. - Citation: *Text contains no information*
- depression_severity: no_depression - LLM Reasoning: Because no depression is present, no severity is indicated; therefore 'no_depression'. - Citation: *Text contains no information*
- depression_severity_mention_context: [] - LLM Reasoning: Without the presence of depression, there is no severity context. - Citation: *Text contains no information*
- depression_status: no_depression - LLM Reasoning: Since no depression exists, there is no current or remitted status; 'no_depression' is applicable. - Citation: *Text contains no information*

Extraction Block: Diagnosis and Cognition

- cognitive_disorder_presence: yes - LLM Reasoning: The diagnosis section explicitly mentions mild dementia, meaning a cognitive disorder is present. - Citation: *Mild dementia with suspected Alzheimer's disease*
- cognitive_disorder_severity: mild - LLM Reasoning: The term "mild dementia" corresponds to the severity "mild" according to the guidelines. - Citation: *Mild dementia with suspected Alzheimer's disease*
- cognitive_disorder_aetiology: [' Alzheimer's Disease] - LLM Reasoning: The text names Alzheimer's disease as the most probable cause of the dementia; other etiologies are not mentioned. - Citation: *The symptom constellation, as well as the slowly progressive course, suggest the presence of Alzheimer's disease as the most likely cause.*
- pathological_amyloid_biomarkers: missing - LLM Reasoning: Neither amyloid CSF values, PET results, nor their pathological evaluation are specified in the entire letter. - Citation: *Text contains no information*

- mini_mental_status_test_result: 26 - LLM Reasoning: The MMSE score is stated as 26 out of 30 points; this value is extracted as the pure score. - Citation: *Mini-Mental-Status Examination 07.03.2023: 26/30 Points (difficulties: spatial orientation 4/5); free Recall 0/3)*

Extraction Block: MRI Findings

- MRI_brain_scan_received: yes - LLM Reasoning: The text explicitly mentions that a cMRI (cranial magnetic resonance imaging) was performed on 10.02.2023. - Citation: *cMRI from 10.2.2023 (external finding): ...*
- MRI_brain_finding_available: yes - LLM Reasoning: A finding of the mentioned MRI is described in the same sentence, meaning a finding is available. - Citation: *cMRI from 10.2.2023 (external finding): Slight patchy white matter gliosis bihemispherical, non-confluent, subcortically accentuated. Global cerebral volume reduction ...*
- MRI_haemorrhage: missing - LLM Reasoning: No signs of bleeding, microbleeds, or explicit statements about the absence of hemorrhages are mentioned in the MRI findings. - Citation: *Text contains no specific information*
- MRI_siderosis: missing - LLM Reasoning: The findings mention neither superficial siderosis nor its exclusion. - Citation: *Text contains no specific information*
- MRI_ischemia: missing - LLM Reasoning: There is no information regarding lacunar or territorial infarcts, neither positive nor excluded. - Citation: *Text contains no specific information*
- MRI_fazekas: missing - LLM Reasoning: The Fazekas score is not mentioned in the findings. - Citation: *Text contains no specific information*
- MRI_cerebral_amyloid_angiopathy: missing - LLM Reasoning: No mention of cerebral amyloid angiopathy or its exclusion. - Citation: *Text contains no specific information*
- MRI_mention_of_other_causes_for_dementia: missing - LLM Reasoning: The findings contain no indications of alternative forms of dementia (e.g., frontotemporal dementia). - Citation: *Text contains no specific information*

Extraction Block: Systemic Diseases and Medication

- immunological_disease: [] - LLM Reasoning: No autoimmune diseases are mentioned in the document; an immunological disease is not explicitly mentioned in the diagnosis, past illnesses, or other findings sections. - Citation: *Text contains no information*
- coagulation_disorder: ['Thrombocytopenia (mild)'] - LLM Reasoning: The findings explicitly mention a " mild-grade thrombocytopenia ", which corresponds to the option "Thrombocytopenia (mild)". - Citation: *Incidental findings showed a mild-grade thrombocytopenia, which should also be checked and co-evaluated by the general practitioner.)*
- **immunosuppression: ['Cortisone'] - LLM Reasoning: Hydrocortisone is a corticosteroid; the category "Cortisone" covers this medication, as it is an immunomodulating steroid. - Citation: *Hydrocortisone ointment as needed***
- anticoagulation: [] - LLM Reasoning: No anticoagulants (e.g., Marcumar, Eliquis, Heparin, etc.) are listed in the text; ASS (Aspirin) is a platelet aggregation inhibitor, but not one of the listed anticoagulants. - Citation: *Text contains no information*
