# Supplementary material for: Locally deployed large language model for real‐world lecanemab eligibility pre‐screening
Source: Alzheimers Dement (Amst). 2026 Sep 27;18(3):e70495. doi: 10.1002/dad2.70495 (PMC13616205; doi:10.1002/dad2.70495)
Supplement: Supplementary file 3 [file DAD2-18-e70495-s003.pdf]

Supplementary Methods1. Deterministic rule-Based Comparator

This supplementary methods document describes the non-LLM rule-based baseline used as a comparator for the LLM-assisted feature extraction pipeline. The baseline was implemented to test whether a compact deterministic extraction approach could replace the LLM extraction layer while keeping the downstream eligibility classifier unchanged.

1. Rule-Based Baseline Algorithm

The rule-based baseline receives the same German memory-clinic consultation reports as the LLM pipeline. It extracts eligibility-relevant intermediate variables using pre-specified regular expressions, small medication and disease dictionaries, local negation handling, family-history filtering, questionnaire-context filtering, and limited numeric parsing for amyloid biomarkers. The extracted variables are then passed to the same deterministic eligibility classifier used for the LLM outputs.

1.1 Extracted variables and classifier interface

The baseline produces the same field names expected by the deterministic classifier. List-valued variables are used for etiologies, medication classes, immunological diseases, coagulation disorders, and anticoagulants. Categorical variables use the same German values as the LLM pipeline, such as ja ("yes"), nein ("no"), fehlt ("missing"), leicht ("mild"), mittel ("moderate"), schwer ("severe"), or a numeric MMSE score.

Domain	Examples of extracted variables	Rule type
**Cognition and diagnosis**	Cognitive impairment, severity, Alzheimer and non-Alzheimer etiologies, MMSE score	Keyword patterns, severity modifiers, score regex
**Amyloid biomarkers**	Pathological, negative, or missing amyloid status	CSF Aß42 terms, Aß42/40 ratio patterns, Amyloid-PET terms, numeric cutoff parsing
**MRI findings**	MRI presence; hemorrhage, siderosis, ischemia, Fazekas 3, CAA	Radiology terms plus negation/exclusion handling
**Clinical history and comorbidity**	Stroke, seizure, depression severity and status	Keyword patterns plus family-history and questionnaire-context filters
**Medication/systemic disease**	Anticoagulants, immunosuppressants, immunological diseases, coagulation disorders	Medication dictionary and disease-term patterns

1.2 Context handling

The algorithm normalizes line breaks, protected spaces, zero-width characters, and typographic dash variants before regex matching. Medication terms are searched both in detected medication sections and across the full report. Candidate concept matches are filtered for local negation and family-history

context; questionnaire-context filtering is applied specifically to depression features. The structured audit workbook stores the study identifier, reference and predicted labels, classifier reasons, extracted feature values, and non-text match summaries; source report text is not included.

- Negation includes pre-concept cues and post-concept exclusion wording, particularly for radiology findings.
- Family-history contexts remove candidate matches referring to relatives rather than the patient.
- For the rule-based extractor, mentions in questionnaire contexts such as NPI-Q and GDS are excluded from depression diagnosis, severity, and status extraction; the LLM uses its own context-sensitive extraction, while both extraction layers feed the same downstream eligibility classifier.
- MMSE extraction requires score anchoring to avoid dates, and supports denominators of 28, 29, or 30.

Amyloid biomarkers are evaluated in line with the eligibility definition and are based on local cut-off scores of the study center: CSF Aß42 values from 0 to 570 are treated as pathologic, Aß42/40 ratio values up to 0.095 are treated as pathologic, and Amyloid-PET is classified from explicit positive or negative wording. For the CSF Aß42 text patterns, low/negative-direction wording such as “decreased” or “reduced” is treated as pathologic, whereas elevated Aß42 wording is not used as positive CSF evidence.

- If several amyloid modalities are mentioned, the rule follows the clinical and/or criterion: any positive CSF Aß42, Aß42/40 ratio, or Amyloid-PET finding is sufficient for a positive amyloid-biomarker variable; only absent positive evidence with negative extracted evidence is classified as negative.

1.3 Representative regular-expression patterns

The following selected patterns document the core rule sets. They are included for reproducibility while avoiding an excessively long appendix. Descriptive labels are translated into English, whereas the regular-expression literals are retained in their original German form to document the implemented rules.

Translation convention: spelling alternatives such as [äa] and German inflection groups such as (?:e|er|en|es)? do not introduce separate concepts; the translations below give each normalized search term.

Selected anticoagulant patterns

('Marcumar (Phenprocoumon)', r'\b(?:Marcumar|phenprocoumon|phenprokumon)\b')

Translations: *Marcumar=Marcumar (brand name); Phenprocoumon/phenprokumon=phenprocoumon.*

('Eliquis (Apixaban)', r'\b(?:Eliquis|apixaban)\b')

Translations: *Eliquis=Eliquis (brand name); Apixaban/apixaban=apixaban.*

('Xarelto (Rivaroxaban)', r'\b(?:Xarelto|rivaroxaban)\b')

Translations: *Xarelto=Xarelto (brand name); Rivaroxaban/rivaroxaban=rivaroxaban.*

('Lixiana (Edoxaban)', r'\b(?:Lixiana|edoxaban)\b')

Translations: *Lixiana=Lixiana (brand name); Edoxaban/edoxaban=edoxaban.*

('Pradaxa (Dabigatran)', r'\b(?:Pradaxa|dabigatran)\b')

Translations: *Pradaxa=Pradaxa (brand name); Dabigatran/dabigatran=dabigatran.*

('Oral anticoagulation', r'\b(?:doak|noak|oak|orale?\s+antikoagulation|antikoagulanzien|antikoagulantien)\b')

Translations: *DOAK=direct oral anticoagulant (DOAC); NOAK=non-vitamin K oral anticoagulant (NOAC); OAK=oral anticoagulation; orale Antikoagulation=oral anticoagulation; Antikoagulanzien/Antikoagulantien=anticoagulants.*

MMSE

MMSE score extraction:

r'\b(?:mmse|mmst|mini[-\s]?mental(?:[-\s]?status)?(?:[-\s]?test)?)\b.{0,90}?\b([0-
3]?\d)\s*(?:/|von)\s*30\s*(?:punkte?|pkt\.?|p\.?)?(?=\W|$)'

__Translations:__ MMSE=Mini-Mental State Examination; MMST=German abbreviation for Mini-Mental State Test; von=of; Punkt/Punkte/Pkt./P.=point(s).

r'\b(?:mmse|mmst|mini[-\s]?mental(?:[-\s]?status)?(?:[-\s]?test)?)\b.{0,90}?\b([0-2]?\d)\s*/\s*(?:28|29)\b'

__Translations:__ MMSE=Mini-Mental State Examination; MMST=German abbreviation for Mini-Mental State Test; remaining expressions are English terms or numeric notation.

r'\b(?:mmse|mmst|mini[-\s]?mental(?:[-\s]?status)?(?:[-\s]?test)?)\s*[:=]\s*([0-3]?\d)(?:\s*[,.;)]|\s*$)'

__Translations:__ MMSE=Mini-Mental State Examination; MMST=German abbreviation for Mini-Mental State Test; remaining expressions are English terms or score punctuation.

Amyloid biomarkers

Reusable helper-pattern definitions (Python; use re.IGNORECASE | re.DOTALL when matching):

import re

ABETA_PREFIX_PATTERN = r'(?:a\s*(?:β|ß|beta)?|abeta|amyloid[-\s]?(?:β|beta)|beta[-\s]?amyloid)'

__Translations:__ Aβ/ABeta/Amyloid-β/Beta-Amyloid=amyloid beta (scientific terms unchanged).

ABETA_42_PATTERN = rf'(?:{ABETA_PREFIX_PATTERN}\s*(?:1[-\s]?)?42)'

__Translations:__ ABETA_PREFIX_PATTERN=amyloid-beta terms translated above; 42=amyloid-beta 42.

ABETA_40_PATTERN = rf'(?:{ABETA_PREFIX_PATTERN}\s*(?:1[-\s]?)?40|(?:1[-\s]?)?40)'

__Translations:__ ABETA_PREFIX_PATTERN=amyloid-beta terms translated above; 40=amyloid-beta 40.

ABETA_RATIO_PATTERN = rf'(?:{ABETA_42_PATTERN}\s*(?:/|zu|:|-)\s*{ABETA_40_PATTERN}|(?:42|1[-
\s]?42)\s*(?:/|zu|:|-)\s*(?:40|1[-\s]?40))'

__Translations:__ zu=to; Aβ42/Aβ40=amyloid-beta 42-to-40 ratio.

CSF_PATTERN = r'(?:liquor|csf|cerebrospinal(?:e|er|en|es)?\s+fluid|nervenwasser)'

__Translations:__ Liquor=cerebrospinal fluid; CSF=cerebrospinal fluid; cerebrospinal(e/er/en/es) fluid=cerebrospinal fluid; Nervenwasser=cerebrospinal fluid.

AMYLOID_LOW_POSITIVE_TERMS_PATTERN =
r'(?:positiv|pathologisch|erniedrigt|vermindert|reduziert|vereinbar|hinweisend|auff[äa]llig|abnorm)'

__Translations:__ positiv=positive; pathologisch=pathological; erniedrigt=decreased; vermindert=reduced; reduziert=reduced; vereinbar=consistent with; hinweisend=indicative of; auffällig=abnormal; abnorm=abnormal.

AMYLOID_PET_PATTERN = r'(?:amyloid[-\s]?pet|amyloid.{0,20}\bpet\b|\bpet\b.{0,20}\bamyloid)'

__Translations:__ Amyloid-PET/PET … Amyloid=amyloid PET (scientific terms unchanged).

AMYLOID_PET_POSITIVE_TERMS_PATTERN =
r'(?:positiv|pathologisch|erh[öo]ht|vermehrt|vereinbar|hinweisend|auff[äa]llig|abnorm)'

__Translations:__ positiv=positive; pathologisch=pathological; erhöht=increased; vermehrt=increased; vereinbar=consistent with; hinweisend=indicative of; auffällig=abnormal; abnorm=abnormal.

CSF Aβ42 text evidence:

CSF_ABETA42_TEXT_EVIDENCE_PATTERN =
rf'\b{CSF_PATTERN}\b.{{0,120}}\b{ABETA_42_PATTERN}\b.{{0,90}}\b{AMYLOID_LOW_POSITIVE_TERMS_P
ATTERN}\b'

__Translations:__ all German search terms are expanded and translated in the referenced helper patterns immediately above.

Aβ42/40 ratio evidence:

ABETA_RATIO_TEXT_EVIDENCE_PATTERN =
rf'\b{ABETA_RATIO_PATTERN}\b.{{0,90}}\b(?:positiv|pathologisch|grenzwertig|auff[äa]llig|erniedrigt|abnorm)\b'

__Translations:__ positiv=positive; pathologisch=pathological; grenzwertig=borderline; auffällig=abnormal; erniedrigt=decreased; abnorm=abnormal; ratio terms are translated above.

Numeric parsing:

CSF Aβ42 ≤ 570 is considered pathological; an Aβ42/40 ratio ≤ 0.095 is considered pathological.

Amyloid PET:

AMYLOID_PET_TEXT_EVIDENCE_PATTERN =
rf'\b{AMYLOID_PET_PATTERN}\b.{{0,90}}\b{AMYLOID_PET_POSITIVE_TERMS_PATTERN}\b'

__Translations:__ all German search terms are expanded and translated in the referenced helper patterns immediately above.

MRI and context filters

MRI presence:
rf'(?<![A-Za-zÄÖÜäöüß])(?:c\s*[-./]?\s*m\s*[-.]?\s*r\s*[-.]?\s*t|m\s*[-.]?\s*r\s*[-.]?\s*t|magnetresonanztomographie|kernspin(?:tomographie)?|sch[äa]del[-\s]?mrt|kopf[-\s]?mrt)(?![A-Za-zÄÖÜäöüß])'

__Translations:__ cMRT=cranial MRI; MRT=MRI; Magnetresonanztomographie=magnetic resonance imaging; Kernspin/Kernspintomographie=magnetic resonance imaging; Schädel-MRT/Kopf-MRT=head MRI.

Hemorrhage:
r'\b(?:h[äa]morrhagie|h[äa]morrhagisch(?:e|er|en|es)?|posth[äa]morrhagisch(?:e|er|en|es)?|blutung(?:en)?|blutungsresid(?:uum|uen)|mikroblutung(?:en)?|microbleed(?:s)?)\b'

__Translations:__ Hämorrhagie=hemorrhage; hämorrhagisch=hemorrhagic; posthämorrhagisch=post-hemorrhagic; Blutung(en)=bleeding/hemorrhage(s); Blutungsresiduum/Blutungsresiduen=residual hemorrhage(s); Mikroblutung(en)=microbleed(s); microbleed(s)=microbleed(s).

Ischemia:
r'\b(?:isch[äa]mie|isch[äa]misch(?:e|er|en|es)?|postisch[äa]misch(?:e|er|en|es)?|infarkt(?:e|areal)?|lakun[äa]r|lakune(?:n)?)\b'

__Translations:__ Ischämie=ischemia; ischämisch=ischemic; postischämisch=post-ischemic; Infarkt(e)/Infarktareal=infarct(s)/infarct area; lakunär=lacunar; Lakune(n)=lacune(s).

Family context:
r'\b(?:familienanamnese|familienanamnestisch|famili[äa]r|familie|mutter|vater|eltern|tochter|sohn|bruder|schwester|gro[ßs]mutter|gro[ßs]vater|oma|opa|tante|onkel|cousine|cousin)\b'

__Translations:__ Familienanamnese=family history; familienanamnestisch=relating to family history; familiär=familial; Familie=family; Mutter=mother; Vater=father; Eltern=parents; Tochter=daughter; Sohn=son; Bruder=brother; Schwester=sister; Großmutter/Oma=grandmother; Großvater/Opa=grandfather; Tante=aunt; Onkel=uncle; Cousine=female cousin; Cousin=male cousin.

Questionnaire context: r'\b(?:npi[-\s]?q|neuropsychiatrisches\s+interview|gds|geriatrische\s+depressionsskala|fragebogen|skala)\b'

__Translations:__ NPI-Q=NPI-Q; neuropsychiatrisches Interview=neuropsychiatric interview; GDS=Geriatric Depression Scale; geriatrische Depressionsskala=Geriatric Depression Scale; Fragebogen=questionnaire; Skala=scale.

1.4 Deterministic eligibility classification

After extraction, the deterministic classifier assigns *not eligible* if a disallowed exclusion criterion is present, including anticoagulation, major MRI exclusion findings, moderate or severe cognitive impairment, negative amyloid biomarkers, MMSE below 22, stroke, epilepsy, or disallowed etiologies/systemic conditions. If no exclusion criterion is present but required positive information is missing, the classifier assigns *potentially eligible*. Only cases with all required positive criteria and no exclusion criterion are classified as *eligible*.

2. Development-Set Refinement Process

The initial rule-based system was constructed as an educated-guess, low-complexity baseline using clinically plausible keywords, medication names, and regular expressions. After each run on the development set, all disagreements between consensus ground truth and the rule-based system were reviewed. Generalizable rule errors were corrected; highly context-dependent cases were not converted into narrow case-specific rules.

2.1 Implemented optimizations after disagreement review

- Restricted short disease abbreviations, including multiple sclerosis and frontotemporal dementia, to avoid non-diagnostic false positives.
- Revised MMSE extraction to avoid dates and to support line breaks, point suffixes, denominators of 28 or 29, and colon-separated MMSE/MMST scores.
- Added local severity parsing for mild thrombocytopenia and thrombocythemia.
- Added full-report fallback matching for anticoagulants and other high-impact medication terms.
- Broadened amyloid biomarker detection to cover CSF Aß42, Aß42/40 ratio, and Amyloid-PET, including numeric cutoff interpretation for CSF Aß42 and the ratio.
- Added family-history filtering for relative-related mentions.
- Improved negation and exclusion handling for radiology findings; broadened MRT/cMRT spelling variants in German reports and prioritized positive MRI evidence over separate non-current or missing-MRI mentions.
- Added hemorrhagic and ischemic sequela vocabulary.
- Mapped early or beginning dementia and Alzheimer-type wording to the corresponding cognitive severity and etiology fields.
- Ignored depression terms occurring only within questionnaire or scale contexts such as NPI-Q and GDS.

2.2 Disagreements not used for rule changes

Five reviewed disagreements did not lead to further rule changes. We considered them limitations that are difficult to solve with compact lexical rules without creating substantial new false positives. They were therefore interpreted as unavoidable limitations of a simple rule-based system.

1. Cognitive severity was expressed with unusual wording describing impairments across several mental domains rather than standard severity terms.
2. Depression severity was mentioned repeatedly, and only broader context clarified that current severity differed from historical severity.
3. A Parkinson syndrome was mentioned, but only broader context clarified that it was a comorbidity and not considered as the cause of dementia.
4. Lewy body dementia was mentioned in an unusual formulation where only broader context made clear that it was excluded.
5. Frontotemporal dementia appeared as part of a specialized clinic name rather than as a patient diagnosis.
