# Supplementary material for: Locally deployed large language model for real‐world lecanemab eligibility pre‐screening
Source: Alzheimers Dement (Amst). 2026 Sep 27;18(3):e70495. doi: 10.1002/dad2.70495 (PMC13616205; doi:10.1002/dad2.70495)
Supplement: Supplementary file 4 [file DAD2-18-e70495-s004.pdf]

Supplementary Table 2. Summary of options given to the large language models for categories with predefined “valid options”

Category	List of Valid options
**Immunological Disease**	"Ankylosing spondylitis (M. Bechterew)", "Crohn's disease (M. Crohn)", “Ulcerative colitis (CU)”, “Psoriatic arthritis (PsA)”, “Rheumatoid arthritis (RA)”, SAPHO syndrome”, “Systemic lupus erythematosus (SLE)”, “Multiple sclerosis (MS)”, “Sjögren’s syndrome”, “Scleroderma”, “Vasculitis”, “Autoimmune hepatitis (AIH)”, “Chronic polyarthritis (cP)”, “Dermatomyositis”
**Bleeding Disorder**	“Thrombophilia”, “Hemophilia A or B”, “Von Willebrand disease (VWD)”, “Thrombocytopenia (mild)”, “Thrombocytopenia (severe)”, “Thrombocythemia (mild)”, “Thrombocythemia (severe)”, "Thrombocytemia vera", “Protein C deficiency”, “Antiphospholipid syndrome (APS)”“Factor V Leiden mutation (FVL)”, “Disseminated intravascular coagulation (DIC)”
**Immunosuppressive Medication**	“Methotrexate (MTX)”, “Cortisone”, “Prednisolone”, “Methylprednisolone”, “Prednisone”, “Dexamethasone”, “Cyclosporine (CSA)”, “Azathioprine (AZA)”, “Tacrolimus”, “Mycophenolate mofetil (MMF)”, “Infliximab”“Adalimumab”, “Etanercept”, “Rituximab”, “Cyclophosphamide (CTX)”, “Oral glucocorticoids”, “Systemic glucocorticoids”
**Anticoagulation**	“Marcumar (Phenprocoumon)”, “Eliquis (Apixaban)”, “Xarelto (Rivaroxaban)”, “Lixiana (Edoxaban)”, “Pradaxa (Dabigatran)”, “Heparin”, “Clexane (Enoxaparin)”, “Warfarin”
