# Supplementary material for: Locally deployed large language model for real‐world lecanemab eligibility pre‐screening
Source: Alzheimers Dement (Amst). 2026 Sep 27;18(3):e70495. doi: 10.1002/dad2.70495 (PMC13616205; doi:10.1002/dad2.70495)
Supplement: Supplementary file 5 [file DAD2-18-e70495-s005.pdf]

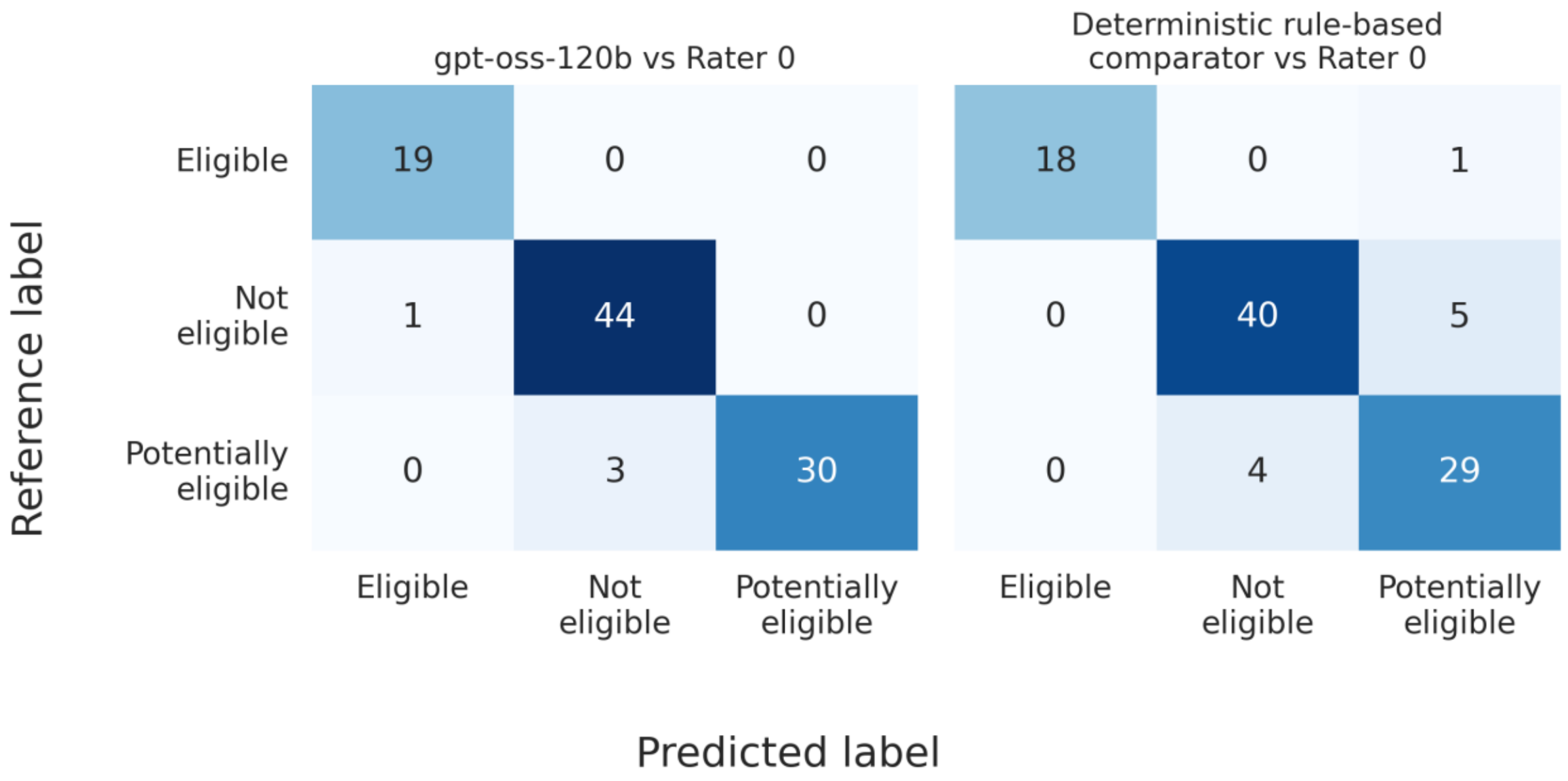

Supplementary Figure 1. Confusion matrices comparing the large language model and deterministic rule-based comparator versus the human gold standard on the development set. Expert annotations (reference labels) are compared with classifications based on the gpt-oss-120b pipeline and deterministic rule-based comparator (predicted labels) on the development set (n=97). Cells along the main diagonal represent matching classifications.
