# Supplementary material for: Locally deployed large language model for real‐world lecanemab eligibility pre‐screening
Source: Alzheimers Dement (Amst). 2026 Sep 27;18(3):e70495. doi: 10.1002/dad2.70495 (PMC13616205; doi:10.1002/dad2.70495)
Supplement: Supplementary file 6 [file DAD2-18-e70495-s006.pdf]

Supplementary Table 3. Comparison of error types observed in the LLM pipeline, rule-based comparator, and human raters

Error type	LLM pipeline	Rule-based comparator	Human raters
**Context misinterpretation**	LBD extracted although ruled out in the clinical context	LBD and NPH interpreted as final diagnoses despite being discussed or ruled out; ruled-out MRI diagnoses interpreted as confirmed; Meningioma interpreted as cause of dementia	–
**Negation failure**	–	Failure to recognize negations (e.g., *no hemorrhage*, *no ischemia*)ndings	–
**Failure to distinguish clinical thresholds**	Fazekas score 1 extracted as contraindication (only Fazekas 3 is a contraindication)	Laboratory reference thresholds misinterpreted as measured amyloid biomarker values;overall amyloid status not inferred from qualitative reports	
**Medication or disease categorization error**	Beclomethasone interpreted as systemic immunosuppressive therapy; psoriasis extracted as immunological disorder	–	–
**Temporal reasoning error**	–	Temporal information misassigned (date of stroke)	–
**Information omission**	–	Missed ischemic MRI findings; severity of cognitive impairment overlooked	MMSE, comorbidities, amyloid biomarkers, or MRI findings overlooked
**Operational definition mismatch**	GDS score prioritized over clinical diagnosis.		Different interpretation of MRI findings
