# Supplementary material for: Locally deployed large language model for real‐world lecanemab eligibility pre‐screening
Source: Alzheimers Dement (Amst). 2026 Sep 27;18(3):e70495. doi: 10.1002/dad2.70495 (PMC13616205; doi:10.1002/dad2.70495)
Supplement: Supplementary file 7 [file DAD2-18-e70495-s007.pdf]

Supplementary Table 1. Summary of instructions and options given to the large language models for each clinical detail

Clinical detail	Instructions and options
**Overall eligibility criteria**	
Cognitive Disorder - Presence	"Yes" if dementia or mild cognitive impairment (MCI), "no" if cognitive impairment is explicitly ruled out, "missing" if dementia or MCI not mentionned
Cognitive Disorder - Severity	"Yes" if mild dementia due to Alzheimer's disease (AD) or mild cognitive impairment (MCI, aMCI), "moderate" if moderate dementia, "severe" if severe dementia, "missing" if no level of cognitive impairment is mentioned, "no cognitive disorder" if category Cognitive disorder - presence = "no"
Cognitive Disorder - Etiology	"Alzheimer's Disease", "Lewy body dementia", "Frontotemporal Dementia (FTD)", "Parkinson's dementia", vascular dementia, "cerebral microangiopathy", "multi-infarct dementia", "progressive supranuclear palsy (PSP)", "normal pressure hydrocephalus (NPH)", "corticobasal degeneration (CBD)", "Huntington's diesease", "primary progressive aphasia (PPA)", "Wernicke encephalopathy", "encephalitis", "tumor", empty list [] if "cognitive disorder - presence" is "no" or "missing"
Biomarker	"Yes" if Aß42 <630 pg/ml)and/or Aß42/40 ratio <0.096 and/or any of these markers is explicitly marked "pathological" and/or if a PET scan detected amyloid, "No" if biomarkers are normal, "missing" if no biomarker data is available
MMSE score	MMSE total score, "-1" if no score available
**MRI eligibility criteria**	
MRI performed	"Yes" if brain MRI was performed, "no" if MRI was not performed, "missing" if MRI not mentioned
MRI report	"Yes" if MRI findings are available and/or explicitly described, "no" if text states that not report is available, "missing" availability of report not expicitly described
MRI haemorrhage	"Yes" if brain hemorrhage (≥10mm) or more than 4 microbleeds mentionned, "no" if findings described as unremarkable with respect to hemorrhage, "missing" if no MRI performed or no statement about hemorrhage
MRI superficial siderosis	"Yes" if superficial siderosis is mentioned, "no" if siderosis explicitly ruled out, "missing" if no MRI performed or siderosis not mentionned
MRI ischemia	"Yes" if > 2 lacunar infarcts or any territorial (including partial) infarcts mentionned, "no" if findings are described as unremarkable with respect to ischemia, "missing" if no MRI performed or no statement about ischemia
MRI Fazekas score	"Yes" if Fazekas score is 3, "no" if Fazekas Score is 0,1 or 2, "missing" if MRI not performed or no statement about fazekas score
MRI cerebral amyloid angiopathy (CAA)	"Yes" if cerebral amyloid angiopathy (CAA), ABRA, or CAA-RI diagnosed in the MRI, "no" if CAA expicitly ruled out, "missing" if no MRI performed or no statement about CAA
**Medical history exclusion criteria**	
History of stroke	"Yes" if stroke within the last 12 months mentioned, "no" if explicitly excluded, "missing" if not mentioned
History of epileptic seizure	"Yes" if epileptic seizure in the past 12 months mentioned, "no" if explicitly excluded, "missing" if not mentioned

History of depression	"Yes" if depressive episode or disorder explicitly mentioned, "no" if explicitly excluded, "missing" if not mentioned
Depression mention context	"Explicit medical diagnosis" if confirmed diagnosis explicitly mentioned, "Treatment (Medication)" if medication mentioned that explicitly targets depressive symptoms, "Treatment (Psychotherapy)" if psychotherapy, "Neuropsychiatric Interview (NPI-Q)" if depression mentioned in the context of the NPI-Q, "Patient Health Questionnaire (PHQ)" if depression is mentioned in the context of the PHQ, "other context" if mentioned in another context, empty list [] if "History of depression" is "no" or "missing"
Depression - Severity	"No depression" if "History of depression" is "no" or "missing", "missing" depression is mentioned but no severity stated, "mild" if mild depression, "moderate" if moderate depression, "severe" if severe/major depression
Depression severity mention context	"Neuropsychiatric Interview (NPI-Q)" if severity is stated in the NPI-Q, "Patient Health Questionnaire (PHQ) if severity is stated in the PHQ, "other context" if severity is stated in another context empty list [] if "History of depression" is "no" or "missing"
Depression - Status	"no depression" if "History of depression" is "no" or "missing", "missing" if depression is mentioned but no status provided, "current" if depression is current at the time of the letter, "remitted" if remission is explicitly mentioned
Immunological disease	One or more of the options listed under "valid options" (Suppl. Table), [exact name] if other immunological disease, if no immunological disease mentioned empty list []
Bleeding disorder	One or more of the options listed under "valid options" (Suppl. Table), [exact name] if other bleeding disorder, if no cogulation disorder mentioned empty list []
**Medication exclusion criteria**	
Immunosuppresive medication	One or more of the options listed under "valid options" (Suppl. Table), [exact name] if medications ending with "-mab", empty list [] if no immunosuppressive medication mentioned
Anticoagulation	One or more of the options listed under "valid options" (Suppl. Table), empty list [] if no anticoagulation mentioned

Abbreviations: AD = Alzheimer's disease, MCI = mild cognitive impairment, aMCI = amnestic mild cognitive impairment, Aß = Amyloid-beta, MMSE = Mini-Mental State Examination, MRI = magnetic resonance imaging, ABRA = Amyloid-beta related angiits, CAA-RI = Cerebral Amyloid Angiopathy related inflammation.
